# Supplementary material for: Identifying Algicides of Enterobacter hormaechei F2 for Control of the Harmful Alga Microcystis aeruginosa
Source: Int J Environ Res Public Health. 2022 Jun 21;19(13):7556. doi: 10.3390/ijerph19137556 (PMC9265343; doi:10.3390/ijerph19137556)
Supplement: Supplementary file 1 [file ijerph-19-07556-s001.zip › Figure S2.pdf]

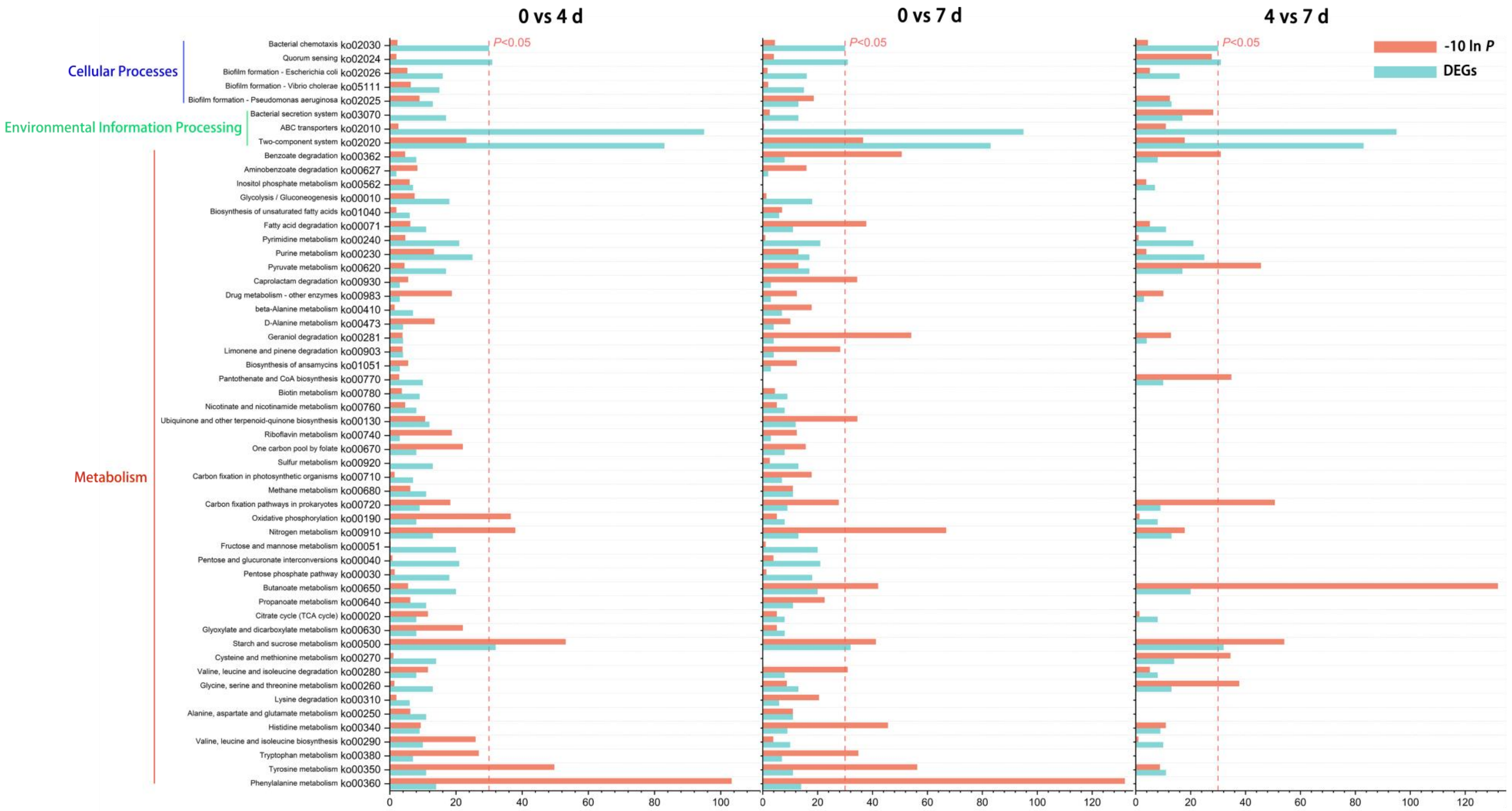

Figure S2. Statistical analysis of differentially expressed genes (DEGs) and Kyoto Encyclopedia of Genes and Genomes (KEGG) functional analysis of DEGs. "*" indicates the significant enrichment of a KEGG pathway (P-value ≤ 0.05)
