# Supplementary material for: Identifying Algicides of Enterobacter hormaechei F2 for Control of the Harmful Alga Microcystis aeruginosa
Source: Int J Environ Res Public Health. 2022 Jun 21;19(13):7556. doi: 10.3390/ijerph19137556 (PMC9265343; doi:10.3390/ijerph19137556)
Supplement: Supplementary file 1 [file ijerph-19-07556-s001.zip › Figure S3.pdf]

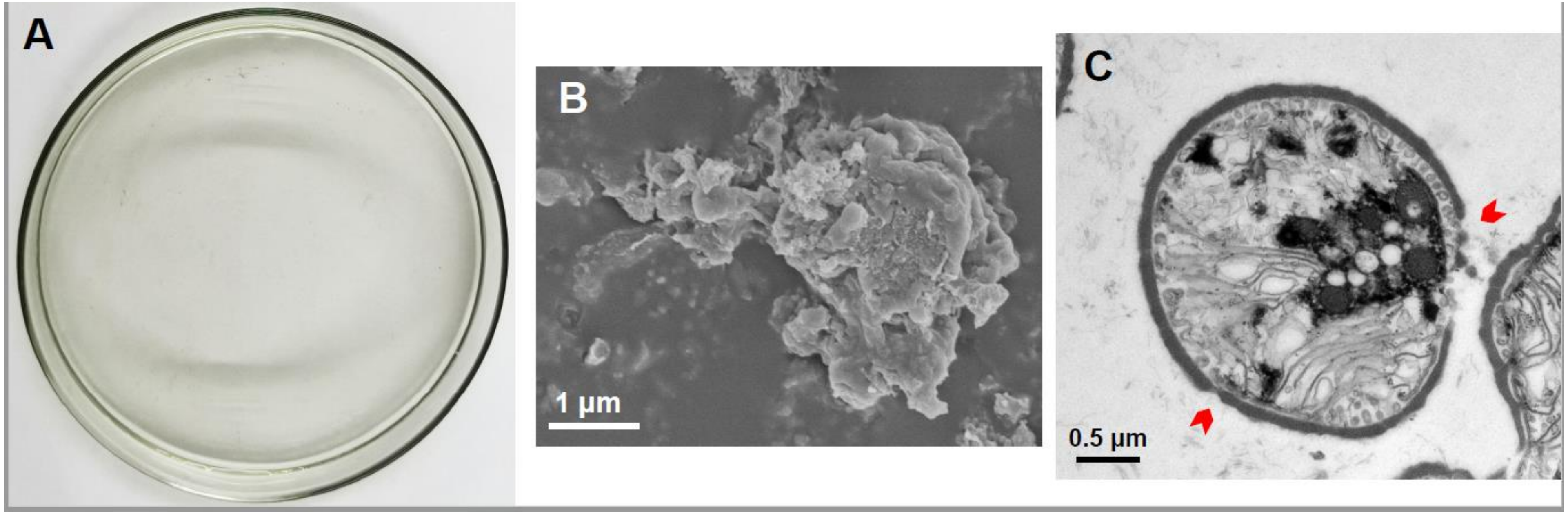


**Figure S3. Algicidal activity of phenazine.** A) Phenotype images; B) scanning electron microscopy images, bar = 1 μm; C) transmission electron microscopy images, bar = 0.5 μm, Algal cell rupture is indicated by red arrows
