# Supplementary material for: Identifying Algicides of Enterobacter hormaechei F2 for Control of the Harmful Alga Microcystis aeruginosa
Source: Int J Environ Res Public Health. 2022 Jun 21;19(13):7556. doi: 10.3390/ijerph19137556 (PMC9265343; doi:10.3390/ijerph19137556)
Supplement: Supplementary file 1 [file ijerph-19-07556-s001.zip › Table S1.pdf]

Table S1A Differentially expressed genes between 0 and 4 d

Gene ID	Gene function	0 d expr	4 d expr
K00652 (CCM2010002799)	8-Amino-7-oxononanoate synthase	49.3497	272.9513
K00100 (CCM2010000041)	Butanol dehydrogenase	216.8151	114.3495
K00540 (CCM2010000625)	Uncharacterized oxidoreductase YhxD	173.9108	43.8759
K00540 (CCM2010001255)	Uncharacterized oxidoreductase YohF	194.9185	112.7173
K00540 (CCM2010001952)	Uncharacterized oxidoreductase YhxD	362.0174	156.6831
K01007 (CCM2010002295)	Phosphonolpyruvate synthase	745.3266	247.932

expr: expression value

Table S1B Differentially expressed genes between 0 d and 7 d

Gene ID	Gene function	0 d expr	7 d expr
K00652 (CCM2010002799)	8-Amino-7-oxononanoate synthase	49.3497	171.7824
K00216 (CCM2010002960)	2,3-Dihydro-2,3-dihydroxybenzoate dehydrogenase	37.2478	139.2999
K00540 (CCM2010000625)	Uncharacterized oxidoreductase YhxD	173.9108	45.5858

expr: expression value
