# Supplementary material for: Identifying Algicides of Enterobacter hormaechei F2 for Control of the Harmful Alga Microcystis aeruginosa
Source: Int J Environ Res Public Health. 2022 Jun 21;19(13):7556. doi: 10.3390/ijerph19137556 (PMC9265343; doi:10.3390/ijerph19137556)
Supplement: Supplementary file 1 [file ijerph-19-07556-s001.zip › Table S2.pdf]

Table S2 Primers used in the study

Primers	Sequences	Gene ID
16S *For*	GCAACGCGAAGAACCTTACCT	16S rDNA
16S *Rev*	GCCATGCAGCACCTGTCT	
K00059 *For*	ACGCAAAATCACCGTCAACT	K00059
K00059 *Rev*	GCGTCACATAGCCTGCTTTA	
K00652 *For*	GGGCGGCGGATGCGTTTC	K00652
K00652 *Rev*	TCTGCGGGTGCTGGCTCAG	
K01657 *For*	CCGCCTCTGCTCGCTCTCC	K01657
K01657 *Rev*	GCCAGCAACCAGGTCGTAAGC	
