# Supplementary figures and images for: Identifying Algicides of Enterobacter hormaechei F2 for Control of the Harmful Alga Microcystis aeruginosa
Source: Int J Environ Res Public Health. 2022 Jun 21;19(13):7556. doi: 10.3390/ijerph19137556 (PMC9265343; doi:10.3390/ijerph19137556)

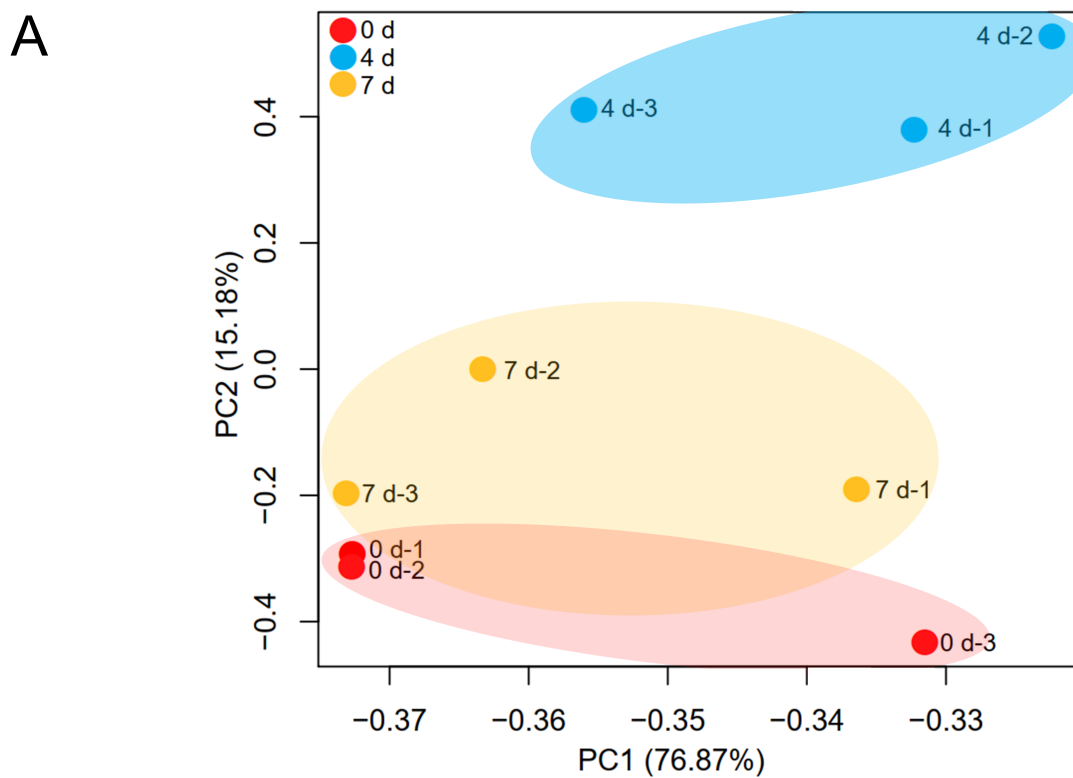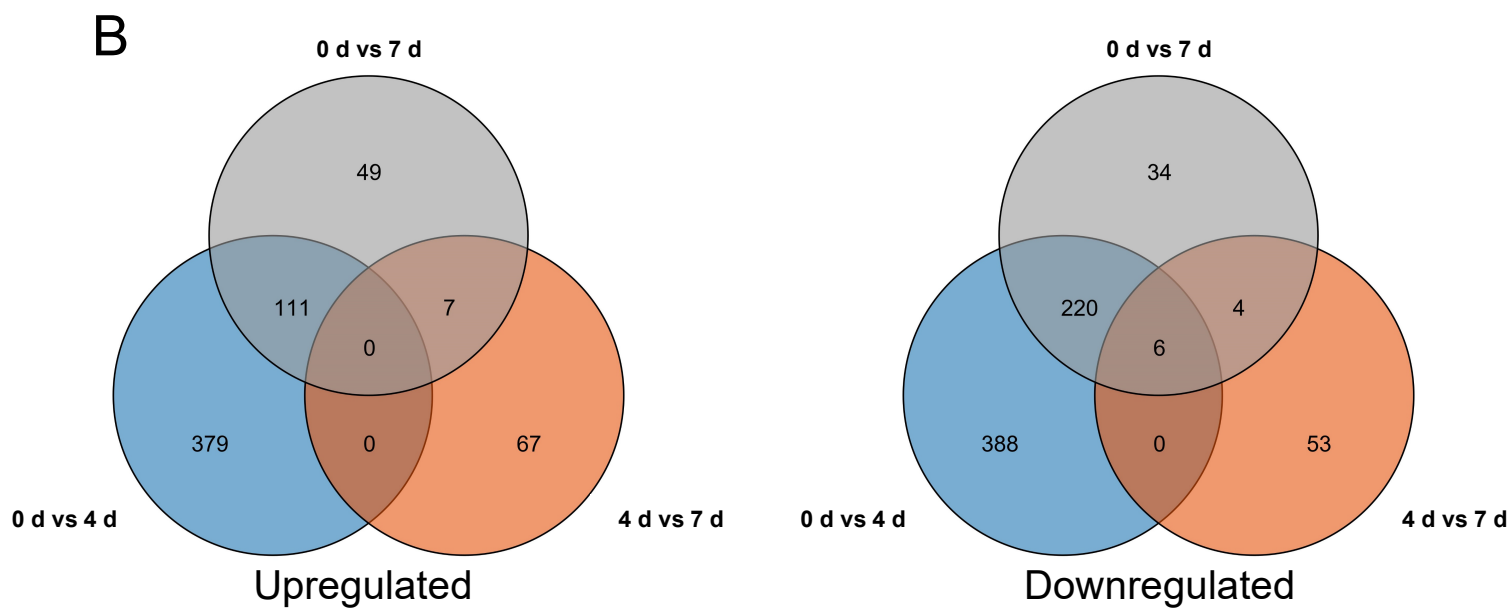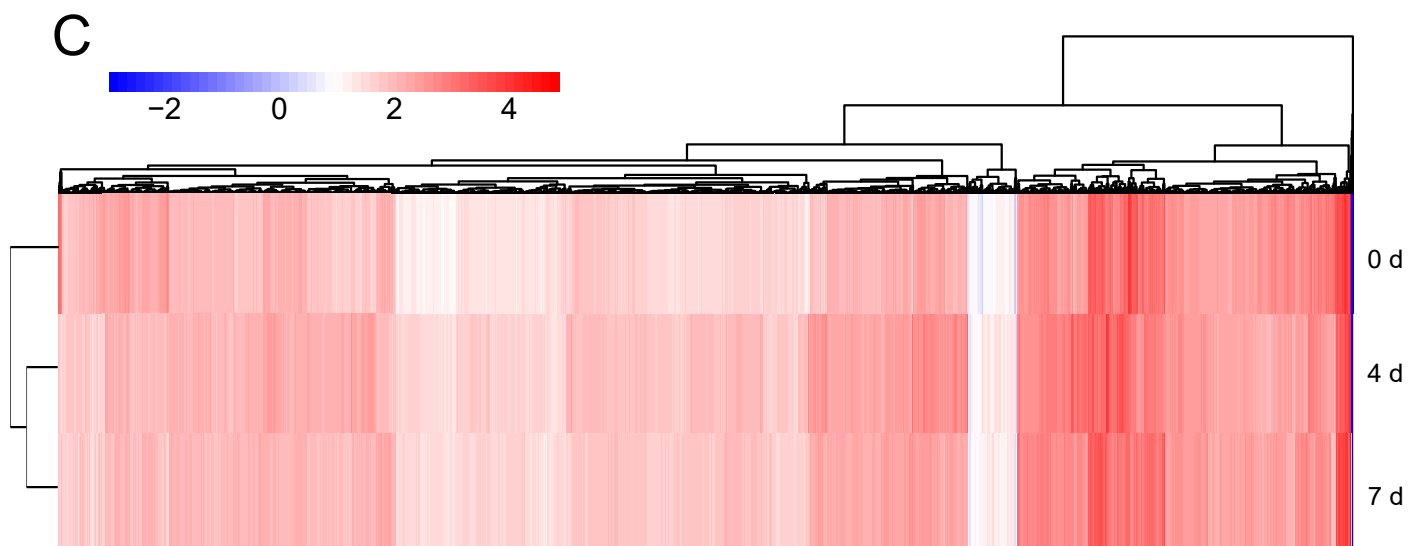

Figure S1. Statistical analysis of differentially expressed genes (DEGs)

Supplement: Supplementary file 1 [file ijerph-19-07556-s001.zip › FigureS1.pdf]
